# BiCAMWI: A Genetic-Based Biclustering Algorithm for Detecting Dynamic Protein Complexes

**DOI:** 10.1371/journal.pone.0159923

**Published:** 2016-07-27

**Authors:** Amir Lakizadeh, Saeed Jalili

**Affiliations:** Computer Engineering Department, Tarbiat Modares University, Tehran, Iran; Koç University, TURKEY

## Abstract

Considering the roles of protein complexes in many biological processes in the cell, detection of protein complexes from available protein-protein interaction (PPI) networks is a key challenge in the post genome era. Despite high dynamicity of cellular systems and dynamic interaction between proteins in a cell, most computational methods have focused on static networks which cannot represent the inherent dynamicity of protein interactions. Recently, some researchers try to exploit the dynamicity of PPI networks by constructing a set of dynamic PPI subnetworks correspondent to each time-point (column) in a gene expression data. However, many genes can participate in multiple biological processes and cellular processes are not necessarily related to every sample, but they might be relevant only for a subset of samples. So, it is more interesting to explore each subnetwork based on a subset of genes and conditions (i.e., biclusters) in a gene expression data. Here, we present a new method, called BiCAMWI to employ dynamicity in detecting protein complexes. The preprocessing phase of the proposed method is based on a novel genetic algorithm that extracts some sets of genes that are co-regulated under some conditions from input gene expression data. Each extracted gene set is called bicluster. In the detection phase of the proposed method, then, based on the biclusters, some dynamic PPI subnetworks are extracted from input static PPI network. Protein complexes are identified by applying a detection method on each dynamic PPI subnetwork and aggregating the results. Experimental results confirm that BiCAMWI effectively models the dynamicity inherent in static PPI networks and achieves significantly better results than state-of-the-art methods. So, we suggest BiCAMWI as a more reliable method for protein complex detection.

## Introduction

In cellular systems, proteins physically interact to form complexes to carry out their biological functions[1, 2]. They are essential building blocks for many biological processes. Therefore, comprehensive investigation of protein complexes from the protein physical interactions can provide a better understanding of basic components and organization of cell machinery, predict protein functions and elucidate cellular mechanisms underlying various diseases from a system level point [3–6]. With recent advance in high-throughput experimental techniques, (e.g. Yeast two-Hybrid (Y2H) and Tandem Affinity Purification with mass spectrometry), large-scale Protein-Protein Interaction (PPI) data has been made available for many species [2, 7, 8]. As a result, One of the most important challenges in the post-genomic era is to analyze these PPIs data and detect protein complexes from them [9]. As a result, over the past decade, many computational methods have been proposed for clustering PPI networks to extract protein complexes from them [10, 11].

According to the absence of temporal information in available physical protein-protein interactions, most computational methods that have been developed during the past decade [10–16] have focused on static networks that have not sufficient data for detecting dynamic protein complexes; however, the cellular processes have dynamic nature and the PPI networks are changing over experimental conditions/times respect to environments and different cell-cycle stages[17–20]. Therefore, it is necessary to shift the analysis of PPI networks from static to dynamic for further understanding of molecular systems [16, 21].

The challenges now are how to employ the dynamic nature of PPI networks and how to detect temporal protein complexes. With recent advance in high-throughput experimental techniques, the massive data from differential expressions of thousands of genes under various experimental conditions/times is provided[22, 23]. If we use gene expression data that are gathered at a sequence of conditions/times during a biological process, we can construct a set of time-sequenced subnetworks. Every subnetwork is an induced subgraph of the original PPI graph that its vertexes are a subset of PPI genes from each time-point; which provides some useful temporal information to complement the static protein interaction data in the gene level. These time-sequenced subnetworks reflect dynamic changes in the original network and provide a dynamic view of most of genes involved in a cellular process and cause to have better understanding of cellular function. More recently, a number of computational methods have focused on integration of PPI networks with time series expression data, to construct time-sequenced subnetworks that exhibit dynamic changes in transcription [24–31].

In some methods [18, 32–34] a threshold is used to determine whether genes are significantly expressed in order to clean the noisy gene expression data. Based on these thresholds, active genes for every time-point are selected. Tang et al.[35] and Li et al. [9] have used a fixed threshold for all time-points of gene expression data. Therefore, if the value of a gene’s expression is greater than the threshold, the gene is assumed to be active otherwise, inactive. In more recent methods [26, 36] authors proposed a distinct threshold for each gene and called it as active threshold. These active thresholds are based on the mean and the standard deviation of a gene expression levels in all time-points.

The above recent methods have a considerable drawback? They completely neglect of the correlations between the subnetworks at successive time-points by merely focusing on the single dynamic PPI subnetworks. However, experimental observations confirm that protein complexes will also formed and carry out their functions dynamically in multiple consecutive conserved across different time-points [31, 32].

To overcome the above shortcomings, more recently, Ou-Yang, et al[17], propose a novel Time Smooth Overlapping Complex Detection model (TS-OCD) to detect overlapping temporal protein complexes from the constructed dynamic PPI subnetworks, which allows individual protein complex to form across different time-points. It is confirmed that protein complexes often have a set of more stably interacting proteins as well as more unstable or transient interactions [37]. TS-OCD can detect both *stable* and *transient* interactions. The stable interactions, as backbone of the protein interaction network are existed through different time-points, while a transient interaction exists at a particular time-point depending on the particular functions two correlated proteins.

Although, TS-OCD is able to distinguish between stable and transient interactions to get higher accuracy, however, an important limitation of TS-OCD and similar methods is the correspondence of each subnetwork to a time-point in gene expression data. In other words, TS-OCD marks an interaction as transient; if the expression level of interaction is more than a certain value. But, many genes can participate in multiple biological processes and cellular processes are not necessarily related to every sample, but they might be correlated only for a subset of samples. So, it is more interesting to extract each subnetwork based on a subset of genes and time-points simultaneously in a time-series gene expression data.

In Clustering, it is possible to cluster rows of gene expression data, however, in cell reality, the genes have same co-regulated and co-expressed patterns only over a subset of experimental samples/conditions and have almost different patterns over the remaining samples/conditions. Such local patterns cannot identify by typical clustering methods. Biclustering methods, provide simultaneous clustering of both rows and columns in the data matrix to discover genes that are co-expressed only in a subset of time-points. Here, biclustering as a powerful tool to discover the biological patterns of co-regulated genes that a clustering algorithm might not recover, give us a better view of the cell dynamic reality.

Biclustering is a NP-Hard problem[38] therefore an exhaustive search for solution space is not feasible. Many biclustering methods apply some heuristics to discover biclusters. Generally, we can classify all of the biclustering methods in two categories: 1) methods based on some evaluation measures and 2) non metric-based methods. In the first category, searching for favorite biclusters is guided by employing a bicluster quality measure (as a cost function). Metric-based methods are divided into four groups: 1) iterative greedy search, 2) stochastic greedy search, 3) nature-inspired meta-heuristics and 4) clustering-based approaches. Most popular metric-based biclustering methods are CC[39], FLOC[40] and MOEA[41]. For a complete survey on bicluster quality measures refer to [42]. Non metric-based methods are divided into five approaches:1) Graph-based, 2) One-way clustering-based, 3) probabilistic models, 4) Linear Algebra and 5) Optimal reordering of rows and columns. Most popular Non metric-based biclustering methods are QUBIC[43], BiMax[44], BBC[45], xMOTISs[46], OPSM[47] and ISA[48]. A more review details on the biclustering algorithms can be found in [49–52]. Moreover, there are some other algorithms introduced to overcome different biclustering problems [53, 54], such as time series gene expression data. Now, biclustering became a powerful tool to discover local patterns on gene expression data [55].

We propose a new method to extract dynamic PPI subnetworks from time series gene expression data. Firstly, it applies a genetic algorithm called GA-DCM to detect biclusters from input gene expression data. GA-DCM uses a novel fitness function called DCM to evaluate biclusters. In comparison with other methods [39, 54, 55], DCM function has two advantages: 1) using a discretized version of gene expression data and bicluster scoring relation in DCM cause to it more robust to the noise that usually there is in expression data, and 2) it can find biclusters with shifting and scaling pattern, while formation of protein complexes in the cell have such shifting and scaling patterns.

Then a post-processing procedure is run to filter out small and not biologically significant biclusters. Next, correspondent to each bicluster, a subgraph consists of the set of genes in bicluster is extracted as a dynamic PPI subnetwork. Similar to TS-OCD, the proposed approach is able to distinguish between stable and transient interactions. Stable interactions, those interactions that exist in all subnetworks (i.e. those are exist across all time-points) while an interaction is transient if its two associated proteins exist at a bicluster. Finally, to assess the effectiveness of this approach, we present a dynamic version of some recent protein complex detection methods. In each case, we run each detection method on all dynamic subnetworks and aggregate all of predicted complexes while removing duplicate ones.

Experimental results show that the proposed dynamicity based on novel biclustering algorithm, can retrieve more significant dynamic subnetworks (means subnetworks that are involved by more protein complexes) from static PPI networks and improves the accuracy of protein complex detection methods. Specially, BiCAMWI, that is a dynamic version of previously presented method, CAMWI[56], achieves significantly better results than TS-OCD and the other state-of-the-art methods. So, we suggest BiCAMWI as a more reliable method for protein complex detection. Source codes of the proposed method are available in S1 File.

## Materials and Methods

**Fig 1:**
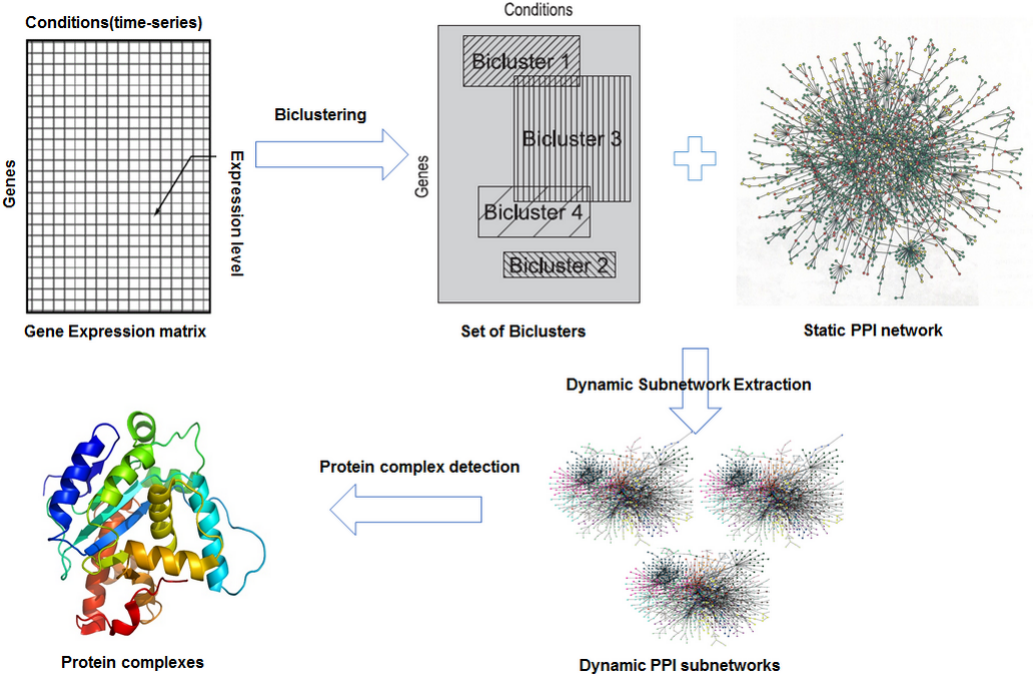
Global view of the proposed method.

This section explains the proposed dynamic method that improves detection accuracy of protein complexes. Our method consists three steps: 1. Develop and applying GA-DCM, a genetic-based biclustering algorithm on gene expression data, to detect biclusters of genes/conditions (subsection 3.1); 2. Extracting dynamic subnetworks from static PPI network based on obtained biclusters (subsection 3.2); and applying a protein complex detection method on every dynamic subnetwork and aggregate the results (subsection 3.3). Fig 1 illustrates the global view of the proposed approach.

### Biclustering algorithm

A genetic algorithm is a metaheuristic tool for solving optimization problems. It simulates the process of natural selection. Genetic algorithm has an iterative procedure. It starts from an initial population of candidate solutions called individuals. Properties (chromosomes) of each candidate solution can be changed. Commonly, the acceptable encoding for each solution is a binary string from 0s and 1s, but other representations are also possible.

In each generation, the goodness of every chromosome is evaluated by a fitness function. According to selection paradigm, the better individuals from the current population are selected and modified by incorporating some genetic operators. The new generation of candidate solutions is now used in the next iteration of the algorithm. Commonly, the algorithm continues to reach a stopping condition. The stopping condition can be either a maximum number of generations, or an adequate fitness level has been reached for the population. Briefly, a traditional genetic algorithm requires: 1) a genetic encoding of the problem solution, 2) a fitness function to evaluate the solutions.

**Fig 2:**
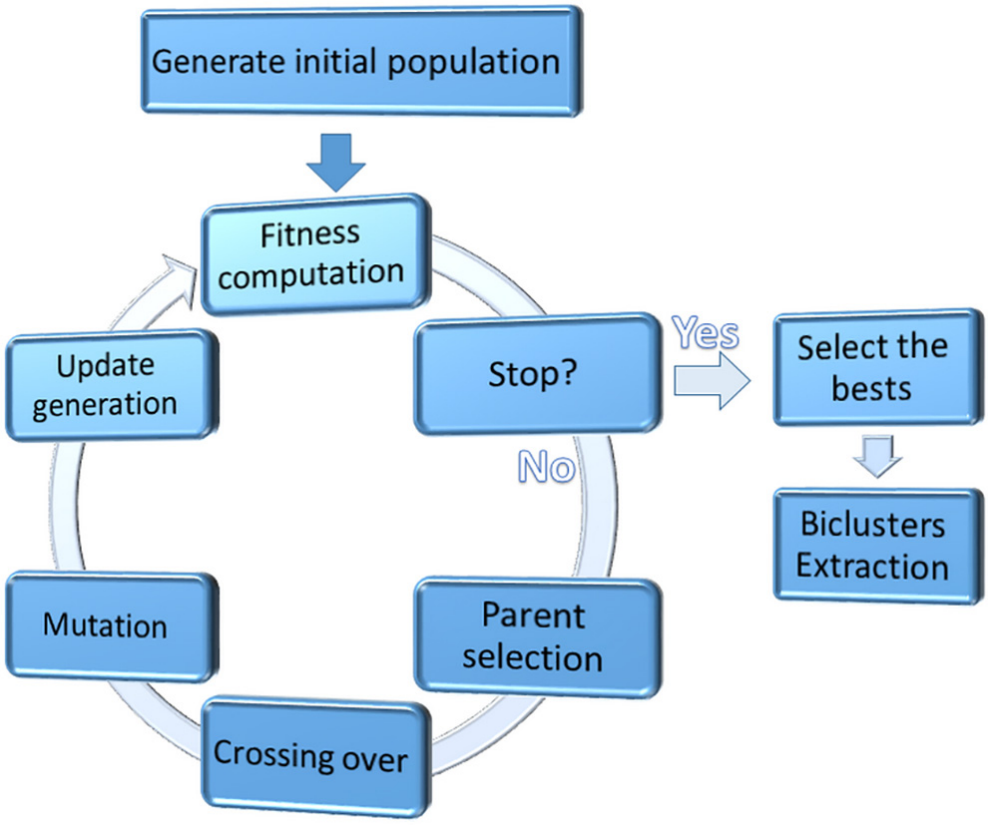
general view of the GA-PCD, the proposed biclustering genetic algorithm.

In this subsection, we explained GA-DCT, the proposed genetic-based biclustering algorithm in details. Encoding of a bicluster of expression matrix into a chromosome of genetic algorithm (3.1.1), introducing of a novel fitness function to measure the quality of a bicluster (3.1.2), and presentation of the genetic operators of the algorithm (3.1.3) are presented in this subsection. Fig 2 shows general flowchart of the proposed genetic algorithm.

#### Bicluster encoding

**Fig 3:**
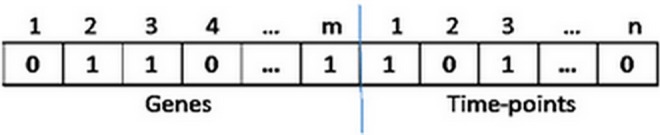
Sample encoding of a bicluster.

Given a two-dimensional gene expression matrix M with m rows and n columns, it contains the expression level of m genes G = {I_1_, I_2_,…, I_m_} over a series of n subsequent time-points(conditions) C = {C_1_, C_2_,…, C_n_} during a biological process. Each element M_i,j_ represents the expression level of the *i*th gene at the *j*th time-point. A bicluster interpreted as a submatrix B(I, J) of expression matrix M, where I, J are subsets of genes set G and conditions set C respectively(I ⊆ G and J ⊆ C). A bicluster is encoded as a genetic chromosome that is represented by a fixed-size binary string composed of genes and time-points. If a gene or condition is included in a bicluster, the corresponding bit is set to 1, otherwise 0. Fig 3 shows an encoding of a bicluster.

#### Fitness function

We perform a preprocessing step before using gene expression data. Preprocessing step consists of two tasks: (i) Data normalization, to make restitution systematical differences between data measured by several microarrays/conditions (the expression matrix M is called M^’^ after performing the normalization step); and (ii) discretization, for reducing the infinite set of real expression values to an acceptable range of discrete values. In normalization step, the expression value of every gene in all time-points are normalized to mean 0 and standard deviation 1. The discretization step provides a number of different discretization techniques replacing each absolute expression value by a symbol of a given alphabet. Alphabets of two or three symbols are the most common, containing the symbols {D, U} and {D, N, U}, respectively, where D means down-regulation, N is no-regulation and U means up-regulation. We consider four discretizing techniques:[57] (i) Simple threshold; (ii) Mean and standard deviation of gene expression profile; (iii) Transitional state discrimination; and (iv) Variation between time-points.

**Simple threshold technique** discretizes expression values in a binary alphabet {D, U} such that if an expression value is higher than the threshold, it is replaced with U Otherwise D.

**Mean and standard deviation of gene expression profile technique** uses an alphabet of three symbols {D, N, U} and parameter *α* defined by the researcher. Symbol D is used to replace all expression values below the difference between the mean value and the product of *α* and the standard deviation. U is used for expression values higher than the sum of the mean value and the product of *α* and the standard deviation. N is used for the remaining expression values.

**Transitional state discrimination** uses a binary alphabet {D, U}. The element M’_i, j_ of the normalized matrix M^’^ is set to U if the difference between M_i, j_ and the M’_i, j_ exceeds 0, otherwise, it set to the symbol D.

**Variation between time-points** can be used in both two and three-letter alphabets. In the binary case, using parameter *α*, a threshold *β* is calculated as the product of *α* and the standard deviation of the expression values of all genes in time-point 0. Then, each element M’_i, j_ of the normalized matrix M^’^ is set to U if the difference between M’_i, j_ and M’_i, j-1_ exceeds the calculated the *β*, Otherwise, it is set to D. In the case of three-letter alphabet, the threshold *β* is directly chosen by researcher. Each element M’_i, j_ of the normalized matrix M^’^ is set to U if the difference between M’_i, j_ and M’_i, j-1_ exceeds *β*, or it is set to D if such difference is lower than—*β*, or N, otherwise.

After choosing the best discretizing technique, we have a discretized expression matrix M”. Here, we define a novel fitness function to determine the quality of biclusters. It is called Discretized Column-based Measure (DCM). Given a bicluster B(I, J) of expression matrix M(G,C), where I ⊆ G and J ⊆ C. The DCM value of B is computed by (Eq (1)).

$${\text{DCM}}_{\text{B}(\text{I},\text{J})}=\sum_{\text{j}\in \text{J}}\left(1\text{-}\frac{(1+\alpha )\times {\text{f}}_{\text{j}}}{\left|\text{I}\right|}\right)$$ (1)

Where α is a penalty factor (0<α<1), |I| is the number of genes in bicluster B and *f*_*j*_ is computed for every column *j* of the bicluster as follows; It counts the frequency of each discrete symbol {D, U}(in case of two-letters alphabets). If a symbol has the majority (means has more than |I|/2 occurrences), then *f*_*j*_ is the number of discretized symbols in column *j* that are unequal to the majority symbol. Otherwise, if none of the symbols have majority, *f*_*j*_ is set to |I|. Also, if the majority symbol is N (in case of three-letters alphabet), *f*_*j*_ is set to |I|/2. Considering (Eq 1), we see that in the best case when all discrete symbols of column j have same value from {D, U}, *f*_*j*_ is 0 and as a result DCM_B(I,J)_ is equal to |J|. On the other hand, in the worst case, if no discrete symbols have majority, DCM_B(I,J)_ is equal to -α×|J|, So, -α×|J| <DCM_B(I,J)_≦ |J|.

#### Genetic operators

Binary tournament[58] is used as selection operator. We design a two-point crossover operator. Crossover is separately performed on both of gene and time-point parts of each chromosome. For the gene part, two crossover points are chosen on each of two selected parent chromosomes, then the segments between these points are exchanged between two parents. For the time-point parts, it is performed similarly. Mutation operator is done as follows. For each bit of selected chromosome (in both gene and time-point parts), it is switched on/off; based on mutation probability.

### Dynamic subnetwork extraction

After constructing biclusters by GA-DCM, the proposed genetic algorithm, with respect to each bicluster, we extract a dynamic subnetwork from input static PPI network. In this step, each subnetwork is an induced sub-graph of the static PPI network that consists of all genes in its corresponding bicluster. In this step, some biclusters may overlap but redundant biclusters are removed before producing dynamic subnetworks.

## Results and Discussion

We assess the proposed dynamic method in three steps. First, we compare GA-DCM with other biclustering algorithms in case of protein complex detection problem. Then, we compared dynamic extension of several protein complex detection methods using the genetic-based biclustering algorithm. The results of this comparison encourage us to suggest BiCAMWI, the dynamic variant of previous proposed method CAMWI[56] as more reliable method for protein complex detection. Finally, we compared BiCAMWI with other detection methods, both static and dynamic methods.

### Datasets and benchmark

To present an accurate analysis, we used two yeast PPI networks to evaluate the efficiency of the compared methods, including: 1) DIP PPI network[59], and 2) BioGrid PPI network (version 3.1.77) [60]. DIP dataset contains 21592 interactions among 4850 proteins, while BioGrid contains 59748 interactions among 5640 proteins. The Yeast Metabolic Cycle (YMC) gene expression microarrays [40] is used to extract significant biclusters and to construct their corresponding dynamic PPI subnetworks. The used expression matrix presents the expression values for 3552 significant periodic genes [61] at 12 time-points (with a time interval about 25 minutes) over three successive cell cycles.

Similar to related researches [17, 26], we used the average expression value of each gene at the same time-point of three cycles. Among 3552 genes, 2389 genes occur in DIP and 3057 genes occur in BioGrid. Thus, we keep these genes and their corresponding interactions in DIP and BioGrid when we prune DIP and BioGrid respectively.

In order to assess the predicted complexes, we use two benchmark experimentally determined complex sets. They are derived from CYC2008 [62] and MIPS [63] respectively. For both gold standard sets, to bypass selection bias, we remove the proteins that are not existed in the two PPI networks. So, it only considered the protein complexes with at least 3 proteins.

### Evaluation measures

f_1_-measure [10] is the most common quality measure which is used for complex detection evaluation. *f*_*1*_*-measure* is used to evaluate the overall performance. It is basically the harmonic mean of two other metrics, precision and recall. precision, measures what fractions of predicted complexes are matched with real complexes. *recall*, is also, what fraction of known complexes are detected correctly.

Let C = {B_1_,B_2_,…,B_n_} denotes the set of benchmark protein complexes and D = {D_1_,D_2_,…,D_m_} denotes the set of detected complexes. To assess protein complex detection, we need to define how well a detected complex matches a known complex. According to the literature, the Jaccard index J(B_i_,D_j_) [11] is used to determine the overlap between a benchmark complex B_i_ and a detected complex D_j_ (Eq (2)).

$$\text{J}({\text{B}}_{\text{i}},{\text{D}}_{\text{j}})=\frac{\left|{\text{B}}_{\text{i}}\cap {\text{D}}_{\text{j}}\right|}{\left|{\text{B}}_{\text{i}}\cup {\text{D}}_{\text{j}}\right|}$$ (2)

Let δ>0 be a predefined similarity threshold. If J(B_i_,D_j_)≥δ then B_i_ and D_j_ are considered as matching complexes. In our study, we set δ = 0.25. precision, recall and f_1_-measure are defined by Eq (3–5) [10, 11].

$$\text{precision}=\frac{\left|\left\{{\text{D}}_{\text{i}}|{\text{D}}_{\text{i}}\in \text{D},\exists \;{\text{B}}_{\text{j}}\in \text{B:J}({\text{D}}_{\text{i}},{\text{B}}_{\text{j}})\geq \delta \right\}\right|}{\left|\text{D}\right|}$$ (3)

$$\text{recall}=\frac{\left|\left\{{\text{B}}_{\text{i}}|{\text{B}}_{\text{i}}\in \text{B},\exists \;{\text{D}}_{\text{j}}\in \text{D:J}({\text{B}}_{\text{i}},{\text{D}}_{\text{j}})\geq \delta \right\}\right|}{\left|\text{B}\right|}$$ (4)

$${\text{f}}_{1}\text{-measure}=\frac{2\times \text{precision}\times \text{recall}}{\text{precision}+\text{recall}}$$ (5)

### Assessment of proposed genetic-based biclustering algorithm

**Fig 4:**
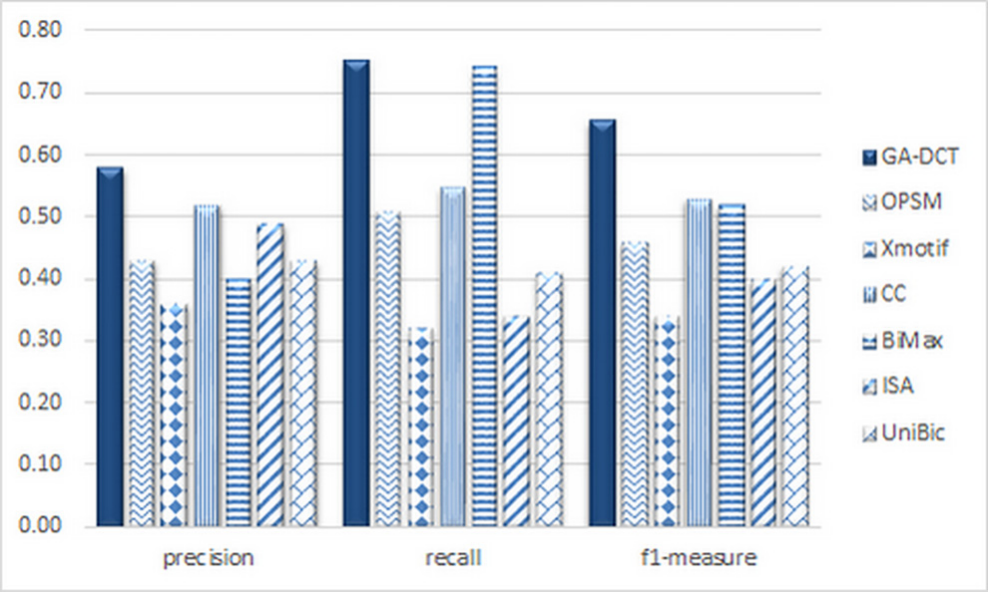
The comparison of GA-DCT with other biclustering algorithms in case of protein complex detection metrics.

It is showed that using a biclustering algorithm to extract some dynamic subnetworks from static PPI networks, improve the accuracy of protein complex detection methods[64]. To have a better view of the proposed genetic-based algorithm advantages, in this subsection, we compare our proposed biclustering method, GA-DCT, with some popular biclustering algorithms CC[39], BiMax[44], xMOTISs[46], OPSM[47],ISA[48] and UniBic[65]. in all methods, after producing biclusters and extracting dynamic subnetworks, CAMWI method[56] is used to detect protein complexes from every dynamic PPI subnetwork. Aggregating the results after removing duplicate complexes produces the final set of detected protein complexes. The comparison results (based on DIP and CYC2008 datasets) in terms of precision, recall and f_1_-measure metrics are showed in Fig 4. Based on the comparison, GA-DCT has better results in terms of all three metrics such that it can retrieve the biclusters that are more consistent to protein complexes nature.

### Accuracy improvement of protein complex detection methods

To show how effectively the proposed method improves the accuracy of protein complex detection methods, we compare the results obtained from running several recent methods, CAMWI[56], COACH [66], ClusterONE [14], SPICi [67] and MCL [68] on three modes: 1) using static variation of methods (with fine-tuning of their parameters), 2) using dynamic variation of methods according to recent dynamic approach presented in TS-OCD, 3) using dynamic variation of methods according to the proposed approach in this paper.

**Fig 5:**
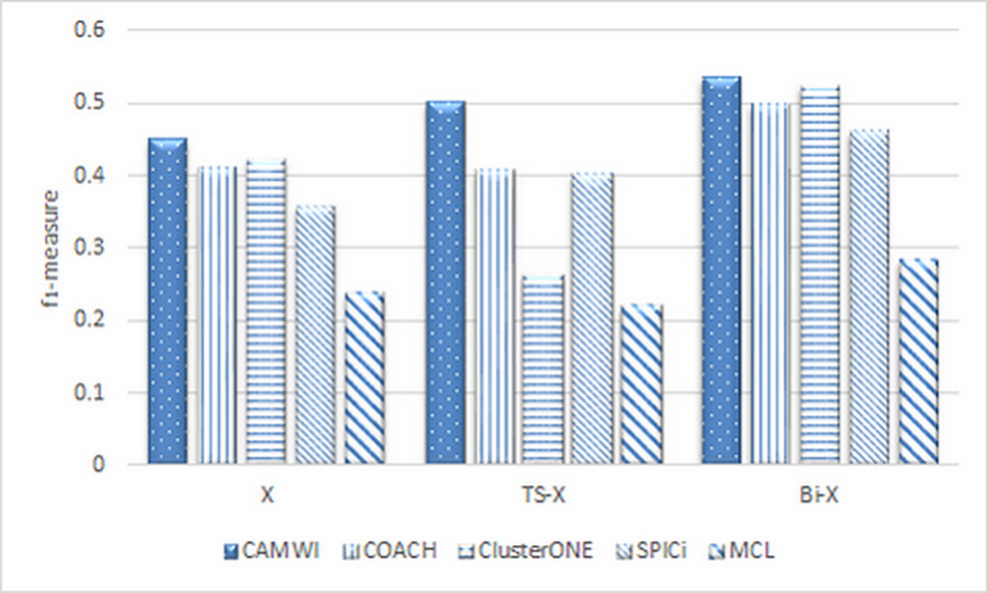
The results of running CAMWI, COACH, ClusterONE, SPICi and MCL methods in term of f1-measure metric. X, TS-X and Bi-X refer to static version, dynamic version of X correspondence to recent dynamic method presented in TS-OCD and dynamic version of X according to GA-DCM dynamic method presented in this paper.

The results in term of *f*_*1*_*-measure* metric based on PPI network Biogrid and CYC2008 benchmark complex set, are shown in Fig 5. For each method i.e. X, we call TS-X and Bi-X to denote dynamic version of X, correspondence to recent dynamic approach presented in TS-OCD and proposed dynamic approach in this paper. Note that in each case, every method is fine-tuned with its best parameters [17].

Experimental results confirm that the proposed genetic-based biclustering approach can use more effectively the inherent dynamicity behind static PPI network such that it improves the accuracy of protein complex detection. Also, these results encourage us to suggest BiCAMWI as a more reliable method for protein complex detection. In the next subsections, we continue the assessment of the proposed approach by comparing BiCAMWI (as the dynamic variant of CAMWI) with other static and dynamic methods.

### The comparison of BiCAMWI with static methods

In order to show how BiCAMWI can effectively detect protein complexes, we compared BiCAMWI method with six state-of-the-art algorithms, namely COACH [66], ClusterONE [14], SPICi [67], MCL [68], MINE [69] and OCD [17]. We ran these methods on static PPI networks DIP and BioGrid. Fig 5 shows the comparative performance on two PPI networks with respect to benchmark complex set CYC2008. Additionally, Table 1 shows details of these comparison in terms of *precision*, *recall* and *f*_*1*_*-measure* metrics for each method on DIP dataset on both benchmark complex sets. Table 2 shows similar facts on BioGrid dataset.

**Table 1:** Analysis details of BiCAMWI comparison with other static methods in terms of precision, recall and f1-measure metrics on DIP dataset and CYC2008 and MIPS benchmark complex sets. Best values are bolded.

	*CYC2008*			*MIPS*		
*Method*	*Precision*	*Recall*	*f1-measure*	*Precision*	*Recall*	*f1-measure*
***ClusterONE***	0.301	0.447	0.36	0.247	0.331	0.283
***SPICi***	0.453	0.417	0.435	0.387	0.331	0.357
***MCL***	0.163	**0.592**	0.255	0.121	0.39	0.185
***COACH***	0.305	0.544	0.39	0.245	0.426	0.311
***MINE***	0.355	0.505	0.417	0.355	0.463	0.402
***OCD***	0.415	0.417	0.416	0.39	0.331	0.358
***CAMWI***	0.43	0.47	0.45	0.35	0.5	0.411
***BiCAMWI***	**0.621**	0.553	**0.585**	**0.607**	**0.514**	**0.556**

**Table 2:** Analysis details of BiCAMWI comparison with other static methods in terms of precision, recall and f1-measure metrics on BioGrid dataset and CYC2008 and MIPS benchmark complex sets. Best values are bolded.

	*CYC2008*			*MIPS*		
*Method*	*Precision*	*Recall*	*f1-measure*	*Precision*	*Recall*	*f1-measure*
*ClusterONE*	0.312	0.655	0.422	0.208	0.445	0.283
*SPICi*	0.294	0.448	0.355	0.228	0.315	0.265
*MCL*	0.167	0.405	0.236	0.133	0.301	0.184
*COACH*	0.324	0.56	0.411	0.247	0.466	0.323
*MINE*	0.311	0.655	0.421	0.201	0.473	0.282
*OCD*	0.332	0.647	0.439	0.27	0.486	0.347
*CAMWI*	0.4	0.61	0.5	0.29	0.5	0.36
*BiCAMWI*	**0.433**	**0.695**	**0.534**	**0.412**	**0.58**	**0.481**

**Fig 6:**
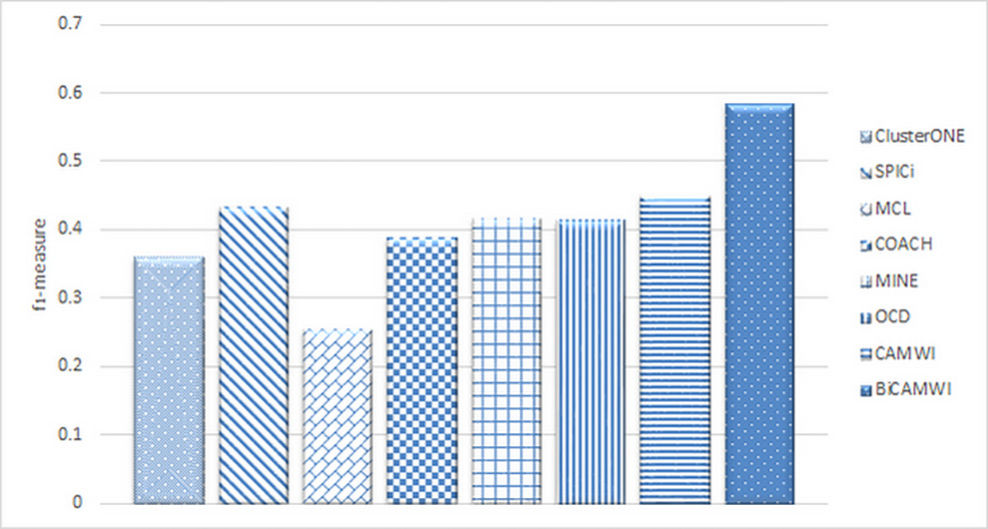
The comparison of BiCAMWI with other static protein complex detection methods. The comparison results are based on DIP dataset in terms of f1-measure with respect to CYC2008 benchmark complex set.

**Fig 7:**
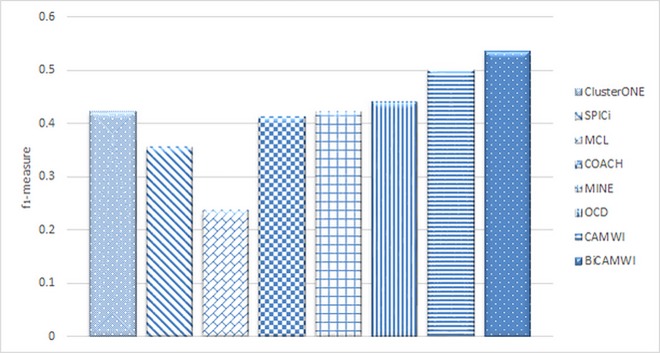
The comparison of BiCAMWI with other static protein complex detection methods. The comparison results are based on BioGrid dataset in terms of f1-measure with respect to CYC2008 benchmark complex set.

The results (Figs 6 and 7) show for both DIP and BioGrid respectively, our BiCAMWI outperforms other methods in terms of *f*_*1*_*-measure* based on CYC2008 benchmark. For instance, on DIP dataset, BiCAMWI achieve the highest *f*_*1*_ -*measure* 0.59, which is 31.1% higher than the second best *f*_*1*_*-measure* 0.45, achieved by CAMWI and %35.6 higher than the third best *f*_*1*_*-measure* achieved by SPICi. On BioGrid dataset, BiCAMWI also achieves the highest *f*_*1*_*-measure* 0.534, which is 9.68% higher than the second best *f*_*1*_*-measure* 0.5 achieved by CAMWI and %21.6 higher than the third best *f*_*1*_*-measure* achieved by OCD.

### The comparison of BiCAMWI with dynamic methods

In another case, we compared BiCAMWI with two recent dynamic methods, Dynamical Hierarchical Agglomerative Clustering (DHAC) method [29] (in two versions: DHAC-local and DHAC-const) and TS-OCD. Also, the result of a dynamic version of COACH, ClusterONE, SPICi, MCL and MINE are from [17]. In all cases, the input gene expression data is used for dynamic considerations. Results in case of DIP and BioGrid datasets and CYC2008 as benchmark are illustrated in Fig 6. Additionally, Table 3 shows details of these comparison in terms of *precision*, *recall* and *f*_*1*_*-measure* metrics on DIP dataset on two benchmark complex sets. Table 4 shows similar facts on BioGrid dataset.

**Table 3:** Analysis details of BiCAMWI comparison with other dynamic methods in terms of precision, recall and f1-measure metrics on DIP dataset divided in CYC2008 and MIPS benchmark complex sets. Best values are bolded.

	*CYC2008*			*MIPS*		
*Method*	*Precision*	*Recall*	*f1-measure*	*Precision*	*Recall*	*f1-measure*
*ClusterONE*	0.327	0.612	0.427	0.299	**0.574**	0.393
*SPICi*	0.369	0.505	0.426	0.342	0.434	0.383
*MCL*	0.17	**0.718**	0.274	0.136	0.544	0.218
*COACH*	0.295	0.553	0.385	0.269	0.5	0.35
*MINE*	0.358	0.592	0.446	0.318	0.566	0.407
*DHAC-const*	0.179	0.184	0.182	0.225	0.154	0.183
*DHAC-local*	0.186	0.0874	0.119	0.274	0.11	0.157
*PCD-GED*	0.485	0.52	0.5	0.45	0.44	0.444
*TS-OCD*	0.429	0.524	0.472	0.397	0.449	0.421
*BiCAMWI*	**0.621**	0.553	**0.585**	**0.567**	0.514	**0.556**

**Table 4:** Analysis details of BiCAMWI comparison with other dynamic methods in terms of precision, recall and f1-measure metrics on BioGrid dataset divided in CYC2008 and MIPS benchmark complex sets. Best values are bolded.

	*CYC2008*			*MIPS*		
*Method*	*Precision*	*Recall*	*f1-measure*	*Precision*	*Recall*	*f1-measure*
*ClusterONE*	0.16	0.724	0.262	0.114	0.479	0.184
*SPICi*	0.285	0.681	0.402	0.255	0.61	0.36
*MCL*	0.134	0.612	0.22	0.0962	0.466	0.159
*COACH*	0.284	0.716	0.406	0.221	0.562	0.317
*MINE*	0.278	**0.741**	0.404	0.234	0.541	0.327
*DHAC-const*	0.413	0.121	0.187	0.413	0.116	0.182
*DHAC-local*	0.323	0.103	0.157	0.362	0.116	0.176
*PCD-GED*	0.43	0.63	0.51	0.35	0.53	0.42
*TS-OCD*	0.363	0.741	0.487	0.312	0.575	0.404
*BiCAMWI*	**0.433**	0.695	**0.534**	**0.412**	**0.58**	**0.481**

**Fig 8:**
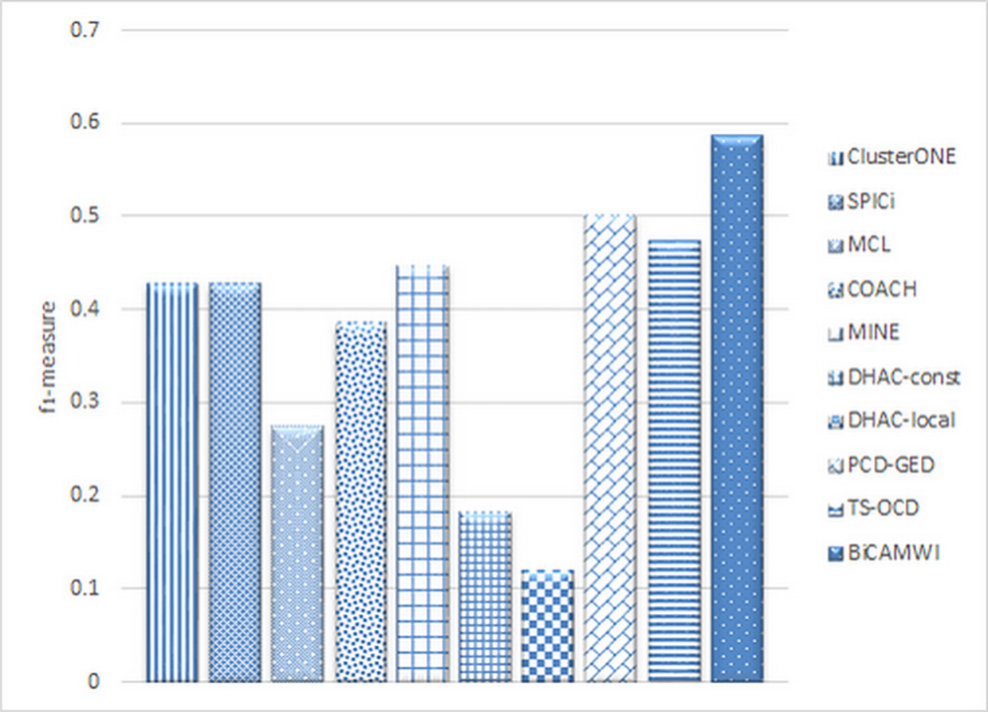
The comparison of BiCAMWI with other dynamic protein complex detection methods. The comparison results are based on DIP dataset in terms of f1-measure with respect to CYC2008 benchmark complex set.

**Fig 9:**
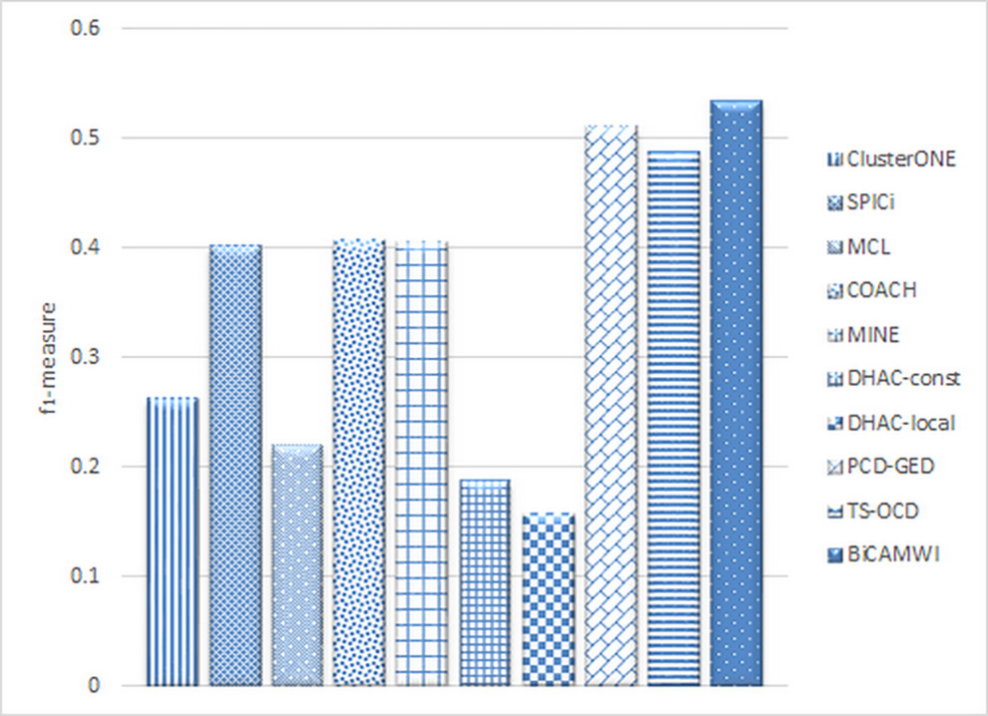
The comparison of BiCAMWI with other dynamic protein complex detection methods. The comparison results are based on BioGrid dataset in terms of f1-measure with respect to CYC2008 benchmark complex set.

The results (Figs 8 and 9) show for both DIP and BioGrid, our BiCAMWI outperforms other methods in terms of *f*_*1*_*-measure* based on CYC2008 benchmark. For instance, on DIP dataset, BiCAMWI achieve the highest *f*_*1*_ -*measure* 0.59, which is 18% higher than the second best *f*_*1*_*-measure* 0.50, achieved by PCD-GED and %25 higher than the third best *f*_*1*_*-measure* achieved by TS-OCD. On BioGrid dataset, BiCAMWI also achieve the highest *f*_*1*_*-measure* 0.534, which is 4.7% higher than the second best *f*_*1*_*-measure* 0.51 achieved by PCD-GED and %9.6 higher than the third best *f*_*1*_*-measure* achieved by TS-OCD.

## Conclusions

Protein complexes are key functional units in many biological processes. Recent advances in high-throughput experimental techniques make large Protein-Protein Interactions (PPIs) networks available. Researchers get PPI networks as input and provide computational methods to detect protein complexes in order to provide a better understanding of basic components and organization of cell machinery. The cellular systems are highly dynamic and protein interaction networks are not static, in other words, they dynamically change across different time-points [17–20, 30]. However, the most computational methods that have been developed during the past decade [10–16] have focused on static PPI networks which cannot model the natural dynamicity of protein interactions.

Recently, a number of methods have concentrated on cell dynamics in computational analysis by integrating PPI networks with time series expression data [24–31]. But these methods try to extract each dynamic subnetwork corresponding to every time-point in gene expression data and completely neglect the correlations between the subnetworks at consecutive time-points. More recent method, TS-OCD [17], that categorizes PPI interactions into stable and transient interactions [37] achieves better results. However, TS-OCD and related researches have an important drawback: they retrieve every dynamic subnetwork correspondent to every time-point in gene expression data and while, genes might be co-regulated and co-expressed only under a subset of experimental conditions, and behave almost independently under other conditions.

It would be more accurate to retrieve some PPI subnetwork based on a subset of genes and time-points (called bicluster) in a time-series gene expression data. Biclustering, which is a two dimensional clustering of both rows and columns in the data matrix, can discover a subset of genes that are co-regulated over a subset of experimental conditions. Here, we proposed a novel method to employ dynamicity in detection of protein complexes. The proposed dynamic method uses a genetic algorithm with a novel fitness function called Discretized Column-based Measure (DCM) to extract significant biclusters from input time-series gene expression data. Then, correspondent to each bicluster, a dynamic PPI subnetwork is extracted from input static PPI network. Final protein complexes set are obtained by performing a detection method on all subnetworks and aggregating the results. The proposed DCM fitness function is based on discretized version of gene expression data. So, it can handle the noise that usually there exists in expression data. Also, it can find biclusters with complicated patterns such as shifting and scaling pattern, while formation of protein complexes in the cell have such patterns. So, it is suitable in case of protein complex detection problem.

Experimental results show that the proposed dynamicity based on biclustering of gene expression data, can retrieve more significant dynamic subnetworks from static PPI networks and improves the accuracy of protein complex detection methods. Specially, BiCAMWI, that is a dynamic variant of previously presented method, CAMWI, achieves significantly better results than TS-OCD and the other state-of-the-art methods. Future works will focus on some improvements on the proposed biclustering algorithm to extract more significant dynamic subnetworks.

## Supporting Information

S1 File Source codes. (RAR)
